# Multiple Concurrent Slotframe Scheduling for Wireless Power Transfer-Enabled Wireless Sensor Networks

**DOI:** 10.3390/s22124520

**Published:** 2022-06-15

**Authors:** Sol-Bee Lee, Sam Nguyen-Xuan, Jung-Hyok Kwon, Eui-Jik Kim

**Affiliations:** 1 Division of Software, Hallym University, 1 Hallymdaehak-gil, Chuncheon 24252, Gangwon-do, Korea; thfqla3535@hallym.ac.kr; 2 Department of Information Technology, Posts and Telecoms Institute of Technology at HCM City Campus, Ho Chi Minh City 71007, Vietnam; samnx@ptithcm.edu.vn; 3 Smart Computing Laboratory, Hallym University, 1 Hallymdaehak-gil, Chuncheon 24252, Gangwon-do, Korea; jhkwon@hallym.ac.kr

**Keywords:** energy harvesting, multiple concurrent slotframes, slotframe length determination, wireless power transfer, wireless sensor network, IEEE 802.15.4 TSCH

## Abstract

This paper presents a multiple concurrent slotframe scheduling (MCSS) protocol for wireless power transfer (WPT)-enabled wireless sensor networks. The MCSS supports a cluster-tree network topology composed of heterogeneous devices, including hybrid access points (HAPs) serving as power transmitting units and sensor nodes serving as power receiving units as well as various types of traffic, such as power, data, and control messages (CMs). To this end, MCSS defines three types of time-slotted channel hopping (TSCH) concurrent slotframes: the CM slotframe, HAP slotframe, and WPT slotframe. These slotframes are used for CM traffic, inter-cluster traffic, and intra-cluster traffic, respectively. In MCSS, the length of each TSCH concurrent slotframe is set to be mutually prime to minimize the overlap between cells allocated in the slotframes, and its transmission priority is determined according to the characteristics of transmitted traffic. In addition, MCSS determines the WPT slotframe length, considering the minimum number of power and data cells required for energy harvesting and data transmission of sensor nodes and the number of overprovisioned cells needed to compensate for overlap between cells. The simulation results demonstrated that MCSS outperforms the legacy TSCH medium access control protocol and TSCH multiple slotframe scheduling (TMSS) for the average end-to-end delay, aggregate throughput, and average harvested energy.

## 1. Introduction

The wireless sensor network (WSN) is an essential technology to provide wireless connectivity between devices in Internet of Things (IoT) systems, serving as a key enabler for implementing various IoT applications, such as smart grid management, process automation, and monitoring [1,2,3,4,5]. In WSNs, sensor nodes require frequent maintenance, such as recharging or replacing the battery, and their communication is often limited when the remaining energy is low because they are generally battery-powered [6,7]. Recently, wireless power transfer (WPT) technology that harvests energy from radio frequency (RF) signals has been widely applied to WSNs; thus, WPT-enabled WSNs that transmit both power and data using an RF signal have the advantage of reducing the battery dependency of sensor nodes and extending the network lifespan [8,9].

Unlike the traditional WSNs, the WPT-enabled WSN includes a hybrid access point (HAP), a device that acts as a power transmitting unit (PTU) to supply power to sensor nodes that act as a power receiving unit (PRU), and includes the existing sensor nodes [10,11].

In WPT-enabled WSN, these heterogeneous devices (i.e., HAPs and sensor nodes) constitute a cluster-tree network, where each cluster includes one HAP serving as a cluster head (CH) and multiple sensor nodes serving as a cluster member [12,13]. Thus, sensor nodes harvest energy from adjacent HAPs and transmit data to the CH. The HAPs collect data from sensor nodes and forward them to the sink. In such a WPT-enabled WSN structure, for timely energy harvesting and data exchange between heterogeneous devices, comprehensively designing a medium access control (MAC) layer protocol considering a network topology and various traffic types (i.e., control messages (CMs), intra-cluster traffic, and inter-cluster traffic) is a challenging issue [14,15,16].

In the literature, many studies have tried to develop the MAC layer protocol for WPT-enabled WSNs. In [17,18,19], the authors proposed carrier sense multiple access with collision avoidance (CSMA/CA)-based MAC protocols, in which HAPs and sensor nodes competitively occupy the channel to perform WPT and data transmission. However, they cause unnecessary energy consumption of sensor nodes and nondeterministic power and data transmission using a random backoff period and retransmission mechanism.

The authors in [20,21] proposed a hybrid MAC protocol that uses a superframe structure, including CSMA/CA and time-division multiple access (TDMA) periods. The use of the CSMA/CA period still causes unnecessary energy consumption of the sensor nodes, while their work combines the benefits of both approaches.

In [22,23,24], the authors proposed TDMA-based harvest-then-transmit protocols, in which energy harvesting and data transmission are performed in a contention-free manner. They allowed sensor nodes to perform energy harvesting and data transmission through frame scheduling, preventing unnecessary energy consumption due to collisions or idle listening. However, the existing TDMA-based harvest-then-transmit protocols may waste resources in WPT-enabled WSNs because they use a fixed-length frame regardless of network topology and traffic type [25].

Time-slotted channel hopping (TSCH) specified in the IEEE 802.15.4-2015 standard [26] can address the problems of the existing MAC protocols by allowing the use of concurrent slotframes. Specifically, TSCH simultaneously uses one or more slotframes with different lengths to efficiently support various communication schedules that vary depending on the network topology and traffic type. Each slotframe consists of multiple cells expressed as a pair with a timeslot and channel offset, and the length of each slotframe is determined according to the number of timeslots. Thus far, several studies have proposed TSCH scheduling schemes using multiple concurrent slotframes to support the network topology and traffic type.

In [27,28,29], the authors employed three types of slotframes. Each slotframe is used for the enhanced beacon (EB), CM, and data. The length of each slotframe is empirically determined, considering the amount of traffic that varies depending on the network topology. However, TSCH scheduling schemes are specialized only for data delivery and use a slotframe structure in which one cell for data transmission and one cell for data reception are allocated within a slotframe based on the sensor node ID. Therefore, their inflexible slotframe structure is unsuitable to support the WPT operation required by the sensor nodes.

In [30], the authors increased the link capacity using a resource allocation algorithm based on the network topology information to support WPT and data transmission in a WSN. However, in [30], a homogeneous network environment composed only of sensor nodes was considered; thus, it is unsuitable for WPT and data transmission operations between heterogeneous devices in WPT-enabled WSNs, including both PTU and PRU.

In [31], the authors proposed a TSCH-based MAC protocol to support energy harvesting and data transmission of sensor devices with different transmission periods. In [31], the sensor devices perform cell allocation using a concurrent slotframe with the same length as their transmission period, causing frequent overlap between cells, and the computational load for solving this increased.

This paper proposes a multiple concurrent slotframe scheduling (MCSS) protocol, supporting a cluster-tree network topology and various traffic types in WPT-enabled WSNs. To this end, MCSS uses three types of TSCH concurrent slotframes: the CM slotframe, HAP slotframe, and WPT slotframe. Each slotframe is dedicated to transmitting the CM traffic, inter-cluster traffic, and intra-cluster traffic. To mitigate the influence of overlapped schedules (i.e., overlapped cells) among slotframes on the network performance, MCSS sets the length of each TSCH concurrent slotframe to be mutually prime and determines the transmission priority of slotframes considering the characteristics of the transmitted traffic. In addition, MCSS determines the WPT slotframe length for each cluster using the numbers of required power and data cells, including the number of overprovisioned cells. The contributions of this work are summarized as follows:

- First, multiple TSCH concurrent slotframes with different lengths are used according to the three traffic types (i.e., CMs traffic, inter-cluster traffic, and intra-cluster traffic) so that MCSS can support a cluster-tree network comprising heterogeneous devices (i.e., HAPs and sensor nodes), which is a general form of the WPT-enabled WSN.
- Second, the WPT slotframe length may be set differently for each cluster based on the amount of intra-cluster traffic, reducing the number of empty cells within the WPT slotframe and improving the channel utilization for energy harvesting and data transmission of sensor nodes.
- Finally, the lengths of TSCH concurrent slotframes are set to be mutually prime to each other, and their transmission priorities are differentiated, thereby, reducing the influence of overlapped cells among the concurrent slotframes.

To verify the superiority of MCSS, we performed an experimental simulation and compared the performance of MCSS with that of the legacy TSCH MAC protocol with a single slotframe. The results demonstrated that MCSS achieved 70.63% and 70.93% shorter end-to-end delay, 17.89% and 18.80% higher aggregate throughput, and 111.96% and 116.28% higher average harvested energy compared with the legacy TSCH MAC and TSCH multiple slotframe scheduling (TMSS), respectively.

The remainder of this paper is organized as follows. Section 2 presents a system model for MCSS. We describe the design of MCSS in detail in Section 3. We provide the simulation configuration and results in Section 4. Finally, Section 5 concludes this paper.

## 2. System Model

In this section, we introduce a system architecture of MCSS in which heterogeneous devices (i.e., HAPs and sensor nodes) constitute a cluster-tree network. Then, we describe the overview of TSCH used as the basic MAC scheme.

### 2.1. System Architecture

**Figure 1:**
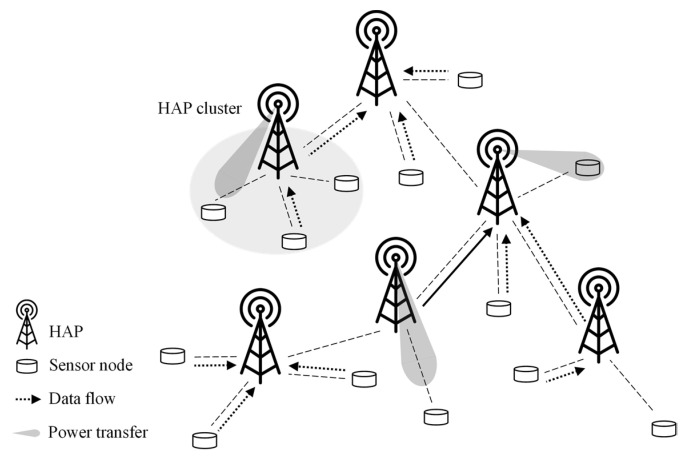
System architecture of MCSS.

Figure 1 illustrates a system architecture of MCSS, consisting of multiple HAPs and sensor nodes. In MCSS, the cluster composed of one HAP and sensor nodes is considered a HAP cluster (i.e., intra-cluster). The HAP is equipped with directional and omnidirectional antennas and acts as a CH in the HAP cluster. It transfers power to adjacent sensor nodes using beamforming technology that steers the beam in a specific direction.

In addition, the HAP transmits the data collected from its cluster members (i.e., sensor nodes) to the neighboring HAP (i.e., parent HAP) so that the collected data can be sent to the root. As HAPs are always connected to a power source, they do not need to harvest energy from any other HAPs; only data are exchanged between HAPs. The sensor node is considered a battery-powered device with a single omnidirectional antenna, harvesting energy from the HAP (i.e., its CH), and transmitting data to the HAP.

In MCSS, the amount of energy consumed by a sensor node to transmit a data packet ($E_{Tx,timeslot}$) is derived based on the energy consumption model in [32] as follows.

(1) $$E_{Tx,timeslot}=\left(P_{sleep}L_{TsTxOffset}+P_{tx}L_{data}+P_{idle}L_{TsRxAckDelay}+P_{rx}L_{Ack}\right)/L_{timeslot},$$

where $P_{sleep}$, $P_{tx}$, $P_{idle}$, and $P_{rx}$ represent the amount of energy consumed when a sensor node is in sleep, transmission (*Tx*), idle, and reception (*Rx*) states during a timeslot, respectively. In addition, $L_{TsTxOffset}$ and $L_{TsRxAckDelay}$ indicate the time intervals for the sensor node to prepare data packet transmission and for the sensor node to wait to receive an acknowledgment (*Ack*) from the HAP, respectively. Further, $L_{data}$, $L_{Ack}$, and $L_{timeslot}$ indicate the time it takes to transmit a data packet, an *Ack*, and the timeslot length, respectively. The HAP supplies power to the sensor nodes based on the amount of energy consumed by each sensor node derived by Equation (1), enabling the data transmission of sensor nodes and preventing their energy depletion.

In MCSS, an IPv6 routing protocol for low-power and lossy networks (RPL) is used for routing between HAPs and sensor nodes [33]. The RPL provides path diversity by organizing a destination-oriented directed acyclic graph (DODAG) according to a routing metric called the *Rank*. The *Rank* refers to the routing distance from a sensor node to the root and is determined by the objective function (OF). The OF of each sensor node computes the *Rank* of each sensor node based on the expected transmission count.

For DODAG formation, each sensor node in RPL broadcasts the DODAG information object (DIO) message containing its *Rank* to its neighbors. Upon receiving the DIO messages, the sensor node selects its parent among the neighbor HAPs considering the *Rank* included in the DIO messages. In MCSS, only HAPs can be selected as the parent of HAPs and sensor nodes. When the parent selection is completed, the sensor node transmits the DODAG advertisement object message toward the root. The root can recognize the new destination information and participation of the sensor node through the received DODAG advertisement object message.

### 2.2. Time-Slotted Channel Hopping

Further, TSCH is a MAC protocol for low-power and lossy networks based on multichannel and time-slotted access standardized in IEEE 802.15.4-2015 [26]. In addition, TSCH supports reliability and deterministic communication between nodes by using a slotframe structure. The slotframe consists of multiple cells and repeats over time. Each cell is identified by the timeslot and channel offset. The cell is long enough to exchange a maximum-size data frame and its *Ack* frame, typically 10 ms long.

The cell can be classified as a dedicated or shared cell. The dedicated cell guarantees transmission between a pair of nodes. In contrast, the shared cell allows multiple nodes to access the same channel simultaneously. In each cell, the nodes communicate on the channel determined by channel hopping as follows:

(2) $$Channel=channelHoppingSequence[(ASN\;+\;offset_{ch})\%\;n_{availableCh}],$$

where the *channelHoppingSequence* represents a list of channels to be hopped; *ASN* denotes the absolute slot number, indicating the total number of timeslots that increase every timeslot since the network started; $offset_{ch}$ refers to the channel offset; and $n_{availableCh}$ indicates the number of available channels in the TSCH network.

In the TSCH network, all nodes are globally synchronized to the network through the EB. The EB includes information, such as the slotframe structure, *ASN*, and *channelHoppingSequence*. After the TSCH network formation is completed, cell allocation is performed. The IPv6 over the TSCH mode of the IEEE 802.15.4e (6TiSCH) operation sublayer (6top) protocol (6P) defined by the IETF 6TiSCH working group provides 6P transaction for TSCH cell allocation [34].

The 6P transaction is a mechanism that enables neighboring nodes to add, delete, and relocate cells to their TSCH schedule. In particular, the 6P transaction can be composed of two or three steps. In a two-step 6P transaction, a candidate cell list consists of cells that can be allocated by the node sending a 6P request message. However, in a three-step 6P transaction, the list contains cells that can be allocated by the node receiving the 6P request message.

**Figure 2:**
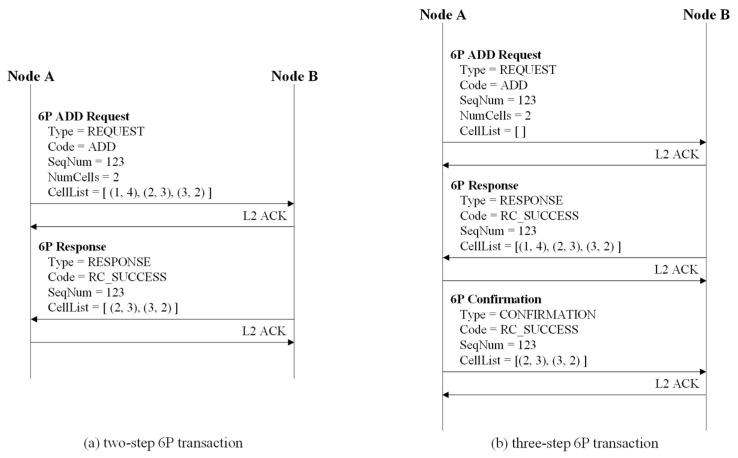
Example of two-step and three-step 6P transactions.

Figure 2 depicts an example of two-step and three-step 6P transactions. In Figure 2a, node A transmits a 6P ADD request message to node B to allocate the required cells. The number of cells to be added (i.e., NumCells), and the list of candidate cells (i.e., CellList) determined by node A are included in the 6P ADD request message. Node B compares the CellList of the received 6P ADD request message to its TSCH schedule to identify which cells are available and selects two available cells. The list of selected cells becomes a CellList to be included in the 6P response message.

Node B informs two selected cells by transmitting the 6P response message to node A. Upon successful completion of the 6P transaction, nodes A and B allocate two selected cells to their TSCH schedule. In Figure 2b, node A transmits a 6P ADD request message to node B, including an empty CellList. When node B receives the 6P ADD request message, node B composes the CellList, including NumCells or more candidate cells available in its TSCH schedule, and transmits the 6P response message to node A.

Node A constructs a CellList by selecting only available candidate cells from the CellList of the 6P response message and transmits a 6P confirmation message containing the CellList to Node B. As a result, the 6P transaction is completed, and nodes A and B allocate the selected cells to their TSCH schedules. In MCSS, we used the three-step 6P transaction to allow each HAP, which knows the schedule (i.e., the results of cell allocation) for all sensor nodes included in the HAP cluster, to construct a candidate cell list.

## 3. Design of MCSS

To support the cluster-tree topology and various types of traffic in WPT-enabled WSN, the MCSS defines multiple TSCH concurrent slotframes, allocates power cells and data cells, and determines the transmission priorities. In the following subsections, we describe the operations of MCSS in detail.

### 3.1. Multiple Concurrent Slotframes

The MCSS uses three slotframes to handle heterogeneous traffic types (i.e., CM traffic, inter-cluster traffic, and intra-cluster traffic) in the WPT-enabled WSN, as follows:

1. The CM slotframe is used to send and receive EB frames, which notify the MCSS schedule of the HAP cluster. It is also used to exchange other CMs, such as RPL messages and 6P messages. In the CM slotframe, one shared cell (TxRxS) capable of both transmission and reception is allocated, through which all CMs are exchanged between neighbors. The length of the CM slotframe is set in consideration of the EB transmission period.
2. The HAP slotframe is used to deliver data traffic (i.e., inter-cluster traffic) between HAPs, for which the HAP allocates at least one data transmission cell (DTx) to transmit data traffic to its parent. In addition, multiple data reception cells (DRxs) can be allocated to the HAP slotframe. The number of data reception cells equals the number of child HAPs of the HAP. The HAP slotframe length is set shorter than other concurrent slotframes to minimize the delivery latency of data traffic.
3. The WPT slotframe supports the transmission of power and data traffic (i.e., intra-cluster traffic) within the HAP cluster. In the WPT slotframe, multiple power transmission cells (PTxs) and data reception cells (DRxs) can be allocated by the HAP and sensor nodes through the three-step 6P transaction described in Section 3.2. The length of the WPT slotframe can be set differently for each HAP cluster and is determined by the HAP.

The MCSS establishes communication schedules by projecting the multiple concurrent slotframes onto a single plane. This operation may cause overlap between cells allocated to different slotframes. To reduce the overlapped cells, MCSS sets the number of timeslots within each slotframe to be a different prime number, making the length of the slotframes mutually prime. In addition, the transmission priority for each slotframe is set to the following priority order considering the traffic characteristics: CM slotframe > HAP slotframe > WPT slotframe.

Accordingly, if overlap occurs between cells, MCSS selects the cells allocated to a slotframe with the highest transmission priority. The WPT slotframe has the lowest transmission priority among slotframes. To compensate for the overlapped cells of the WPT slotframe, MCSS determines the length of the WPT slotframe for each HAP cluster using the minimum number of power and data cells and the number of overprovisioned cells. The length determination for the WPT slotframe is described in Section 3.2 in detail.

**Figure 3:**
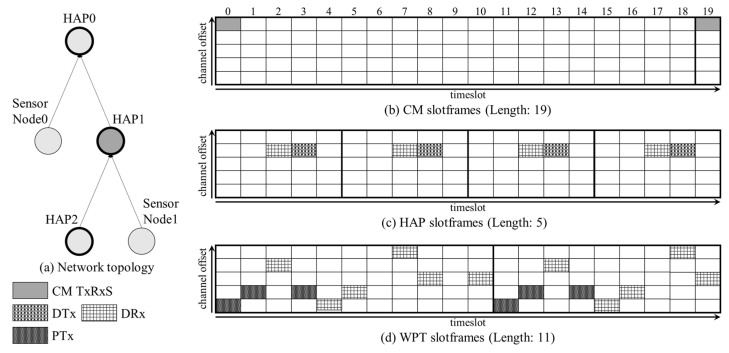
Example of multiple concurrent slotframes in MCSS.

Figure 3a presents an example of a WPT-enabled WSN topology consisting of three HAPs (HAP 0, 1, and 2) and two sensor nodes (sensor node 0 and 1). Figure 3b–d represent the CM, HAP, and WPT slotframes, and their lengths are 19, 5, and 11, respectively. In addition, HAP 1 can broadcast its EB frame in timeslots 0 and 19 (CM TxRxS) within the CM slotframes.

In addition, it can exchange the RPL and 6P messages with its parent (i.e., HAP 0) or child nodes (i.e., HAP 2 and sensor node 1) in timeslots 0 and 19. Moreover, HAP 1 receives data packets from HAP 2 in timeslots 2, 7, 12, and 17 (DRxs) and transmits data packets to HAP 0 in timeslots 3, 8, 13, and 18 (DTxs) within the HAP slotframes. In the WPT slotframe, HAP 1 transmits power to sensor node 1 using power transmission cells (PTxs) and receives data packets from sensor node 1 using data reception cells (DRxs).

**Figure 4:**
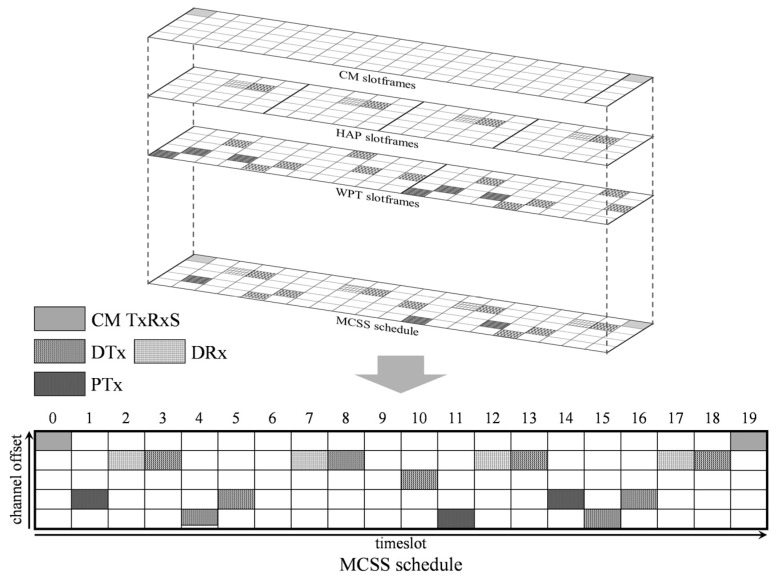
Example of an MCSS schedule.

Figure 4 illustrates the MCSS schedule of HAP 1 in Figure 3a. In the figure, multiple concurrent slotframes of HAP 1 are projected onto one plane, and as a result, only one cell is allocated to each timeslot. Specifically, the data reception cell (DRx) of the HAP slotframe and data reception cell (DRx) of the WPT slotframe overlap in the cell (1 and 2) of the MCSS schedule. In this case, the data reception cell (DRx) of the HAP slotframe is allocated to the MCSS schedule of HAP 1 because the HAP slotframe has a higher priority than the WPT slotframe. At timeslots 0, 2, 3, 7, 8, 12, 13, 18, and 19, two or more cells with different channel offsets are scheduled; however, only one cell is allocated to the MCSS schedule according to the priority of concurrent slotframes.

### 3.2. Length Determination and Cell Allocation for WPT Slotframe

In WPT-enabled WSNs, the amount of intra-cluster traffic for each HAP cluster depends on the number of cluster members. Therefore, MCSS determines the WPT slotframe length differently for each HAP cluster, considering the minimum number of power and data cells required for energy harvesting and data transmission of cluster members and the number of overlapped cells among slotframes. To allocate power and data cells to the WPT slotframe, MCSS uses the three-step 6P transaction specified in the 6TiSCH standard [34]. However, unlike the existing standard, MCSS uses the modified 6P messages to accommodate all information for power cell allocation and data cell allocation.

To determine the WPT slotframe length, the HAP broadcasts the EB frame, including the length of the initial WPT slotframe. We assume that the initial WPT slotframe length is the same as the default slotframe length in the minimal 6TiSCH configuration [35]. Then, the HAP waits until it receives hello messages from all cluster members in the HAP cluster. The hello message includes the minimum number of data cells, which should be allocated to the initial WPT slotframe to accommodate the data traffic transmitted from the cluster member. Upon receiving the message from all cluster members, the HAP calculates the minimum number of power cells using the minimum number of data cells. The minimum number of power cells required for the *i*-th cluster member ($n_{minPC,i}$) is calculated as follows:

(3) $$n_{minPC,i}=n_{minDC,i}\left\lceil \frac{E_{Tx,timeslot}}{E_{Rx,timeslot,i}}\right\rceil \;\;\;(0<i\leq n_{node,j},\;0<j\leq M),$$

where *i* refers to the index of the cluster member in the HAP cluster, *j* denotes the index of the HAP, and $n_{node,j}$ is the number of cluster members for the *j*-th HAP cluster. Moreover, *M* represents the total number of HAPs in the network, $n_{minDC,i}$ denotes the minimum number of data cells required for the *i*-th cluster member, and $E_{Rx,timeslot,i}$ refers to the energy received by the *i*-th cluster member from the HAP (i.e., its parent) during one timeslot. Finally, $E_{Rx,timeslot,i}$ is calculated as follows:

(4) $$E_{Rx,timeslot,i}=P_{Rx,i}L_{timeslot}\;\;\;(0<i\leq n_{node,j},\;0<j\leq M),$$

where $P_{Rx,i}$ represents the power that the *i*-th cluster member receives per second. In addition, $P_{Rx,i}$ is calculated as follows:

(5) $$P_{Rx,i}=\eta P_{Tx}{\left|h\right|}^{2}=\eta P_{Tx}/(1+{\left|d_{i}\right|}^{\alpha })\;\;\;(0<i\leq n_{node,j},\;0<j\leq M),$$

where η refers to the energy harvesting efficiency factor, $P_{Tx}$ represents the transmission power of the HAP, *h* denotes the harvested power gain from the HAP, $d_{i}$ indicates the distance between the *i*-th cluster member and its parent, and α refers to the path-loss parameter. Moreover, $d_{i}$ is calculated as follows.

(6) $$d_{i}=10^{\frac{-rssi_{avg,i}+A}{10\alpha }}\;\;\;(0<i\leq n_{node,j},\;0<j\leq M)$$

where *A* refers to the received signal strength of the cluster member at a distance of 1 m from its parent, and $rssi_{avg,i}$ denotes the average received signal strength indicator (RSSI) of data packets received from the parent of the *i*-th cluster member.

Then, the HAP calculates the numbers of overprovisioned power and data cells, which refer to power and data cells that must be allocated to compensate for the overlap between cells. The allocation of overprovisioned cells prevents power and data traffic delays caused by a difference in priority between slotframes. The numbers of overprovisioned power and data cells for the *i*-th cluster member ($n_{overPC,i}$ and $n_{overDC,i}$) are calculated by considering the ratio of $n_{minPC,i}$ and $n_{minDC,i}$ to the sum of the minimum numbers of the power and data cells for all cluster members in the *j*-th HAP cluster, expressed as follows:

(7) $$n_{overPC,i}=round\left(\frac{n_{overcell,j}n_{minPC,i}}{\sum_{i=1}^{n_{node,j}}\left(n_{minPC,i}+n_{minDC,i}\right)}\right)\;\;\;(0<i\leq n_{node,j},\;0<j\leq M),$$

(8) $$n_{overDC,i}=round\left(\frac{n_{overcell,j}n_{minDC,i}}{\sum_{i=1}^{n_{node,j}}\left(n_{minPC,i}+n_{minDC,i}\right)}\right)\;\;\;(0<i\leq n_{node,j},\;0<j\leq M),$$

where $n_{overcell,j}$ represents the total number of overprovisioned cells in the *j*-th HAP cluster. Further, $n_{overcell,j}$ is determined based on the least common multiple (LCM) of multiple concurrent slotframes of the *j*-th HAP cluster ($l_{LCM,j}$), calculated as follows:

(9) $$n_{overcell,j}=\left\lfloor \frac{l_{WPT,j}n_{totalcell,j}}{l_{LCM,j}n_{node,j}}\right\rfloor \;\;\;(0<j\leq M),$$

where $l_{WPT,j}$ represents the initial WPT slotframe length for the *j*-th HAP cluster. In addition, $n_{totalcell,j}$ denotes the total number of cells expected to be allocated to the MCSS schedule for the *j*-th HAP cluster during $l_{LCM,j}$ timeslots, calculated as follows:

(10) $$n_{totalcell,j}=n_{CMcell,j}+n_{HAPcell,j}-n_{dupcell,CM\&HAP}{}_{,j}\;\;\;(0<j\leq M)$$

where $n_{CMcell,j}$ and $n_{HAPcell,j}$ are the respective numbers of cells expected to be independently allocated to the CM and HAP slotframes of the *j*-th HAP cluster during $l_{LCM,j}$ timeslots. Additionally, $n_{dupcell,CM\&HAP}{}_{,j}$ is the number of cells expected to be redundantly allocated between the CM and HAP slotframes in the *j*-th HAP cluster during $l_{LCM,j}$ timeslots. Moreover, $n_{CMcell,j}$ and $n_{HAPcell,j}$ in Equation (10) are calculated as follows:

(11) $$n_{CMcell,j}=\frac{l_{LCM,j}}{L_{CM}}\;\;\;(0<j\leq M),$$

(12) $$n_{HAPcell,j}=\frac{l_{LCM,j}n_{HAP,j}}{L_{HAP}}\;\;\;(0<j\leq M),$$

where $L_{CM}$ and $L_{HAP}$ denote the length of CM and HAP slotframes, respectively. The term $n_{HAP,j}$ refers to the total number of HAPs in the *j*-th HAP cluster. In addition, $n_{dupcell,CM\&HAP}{}_{,j}$ in Equation (10) is determined based on the relationship between repeated cycles of multiple concurrent slotframes for the same period, calculated as follows:

(13) $$n_{dupcell,CM\&HAP}{}_{,j}=\frac{l_{LCM,j}n_{HAP,j}}{LCM(L_{CM},L_{HAP})}\;\;\;(0<j\leq M)$$

where $LCM(L_{CM},L_{HAP})$ represents the LCM of the lengths of the CM and HAP slotframes. Furthermore, $LCM(x_{1},x_{2},\cdots ,x_{w})$ refers to the LCM of $x_{1}$, $x_{2}$, …, $x_{w}$, where *w* is the number of multiple concurrent slotframes.

Consequently, the numbers of required power and data cells for the *i*-th cluster member in the HAP cluster ($n_{reqPC,i}$ and $n_{reqDC,i}$) are calculated as follows:

(14) $$n_{reqPC,i}=n_{minPC,i}+n_{overPC,i}\;\;\;(0<i\leq n_{node,j},\;0<j\leq M),$$

(15) $$n_{reqDC,i}=n_{minDC,i}+n_{overDC,i}\;\;\;(0<i\leq n_{node,j},\;0<j\leq M).$$

For the WPT slotframe length determination, each HAP maintains a HAP cluster table (HCT) containing information about the cluster members. Specifically, the table entry of the HCT for the *j*-th HAP (i.e., the *j*-th HCT) includes the ID list ($ID_{\left(j\right)}$) and numbers of required power and data cell lists ($N_{reqPC(j)}$ and $N_{reqDC(j)}$) of its cluster members of the *j*-th HAP. Finally, $ID_{\left(j\right)}$, $N_{reqPC(j)}$, and $N_{reqDC(j)}$ are represented as follows:

(16) $$ID_{\left(j\right)}=\left[id_{\left(1\right)},id_{\left(2\right)},\cdots ,id_{\left(i\right)}\right],\;\;\;(0<i\leq n_{node,j},\;0<j\leq M),$$

(17) $$N_{reqPC(j)}=\left[n_{reqPC,1},n_{reqPC,2},\cdots ,n_{reqPC}{}_{,i}\right],\;\;\;(0<i\leq n_{node,j},\;0<j\leq M),\;\mathrm{and}$$

(18) $$N_{reqDC(j)}=\left[n_{reqDC,1},n_{reqDC,2},\cdots ,n_{reqDC,i}\right],\;\;\;(0<i\leq n_{node,j},\;0<j\leq M),$$

where $id_{\left(i\right)}$ indicates ID of the *i*-th cluster member in the *j*-th HAP cluster.

Algorithm 1 presents the procedure of the WPT slotframe length determination for each HAP. The HAP initializes variables (i.e., $l_{minWPT,j}$ and primeNumFlag), and $l_{minWPT,j}$ represents the minimum length required to determine the length of the WPT slotframe for the *j*-th HAP cluster, which is the sum of $n_{reqPC,i}$ and $n_{reqDC,i}$ for all cluster members in the HCT. In addition, primeNumFlag determines whether the HAP locates the smallest prime number greater than $l_{minWPT,j}$. Then, the HAP calculates $l_{minWPT,j}$ by adding $n_{reqPC,i}$ and $n_{reqDC,i}$ for all cluster members included in $ID_{\left(j\right)}$. Moreover, $l_{minWPT,j}$ of the *j*-th HAP, $l_{minWPT,j}$, is calculated as follows:

(19) $$l_{\min WPT,j}=\sum_{k=0}^{n_{node,j}-1}\left(n_{reqPC,k}+n_{reqDC,k}\right),\;\;\;0\leq k\leq n_{node,j}-1.$$

If $l_{minWPT,j}$ is not a prime number, the *j*-th HAP increases $l_{minWPT,j}$ by increments of 1 to determine the smallest prime number greater than $l_{minWPT,j}$. Finally, the HAP sets $l_{WPT,j}$ to the determined $l_{minWPT,j}$. After the length determination for the WPT slotframe is completed, the HAP broadcasts the EB frame to notify its cluster members of $l_{WPT,j}$.
**Algorithm 1.** WPT slotframe length determination1:**INITIALIZE** primeNumFlag to TRUE, $l_{minWPT,j}$ to 02:**FOR** $id_{\left(i\right)}$, i, $i\in \left[0,n_{node,j}\right]$3:    
$l_{minWPT,j}\leftarrow l_{minWPT,j}+n_{reqPC,i}+n_{reqDC,i}$
4:**ENDFOR**5:**WHILE**primeNumFlag6:    **FOR** each iteration, k, $j\in \left[2,\mathrm{math}.\mathrm{sqrt}(l_{minWPT,j})+1\right]$
7:        **IF** $l_{minWPT,j}\;\%\;k==0$8:            primeNumFlag←TRUE9:            $l_{minWPT,j}=l_{minWPT,j}+1$
10:            Break11:        **ELSE**
12:            primeNumFlag←FALSE13:        **ENDIF**
14:    **ENDFOR**
15:**ENDWHILE**16:$l_{WPT,j}\leftarrow l_{minWPT,j}$17:**RETURN** 
$l_{WPT,j}$


Upon receiving the EB frame, including the numbers of required power and data cells ($n_{reqPC,i}$ and $n_{reqDC,i}$), the cluster member initiates the three-step 6P transaction to allocate power and data cells to the WPT slotframe. To this end, the *i*-th cluster member transmits the modified 6P ADD request, including $n_{reqPC,i}$ and $n_{reqDC,i}$ to its parent along with the empty PowerCellList and empty DataCellList. When the HAP receives the modified 6P ADD request from the *i*-th cluster member in the HAP cluster, it configures the PowerCellList and DataCellList for the *i*-th cluster member ($PC_{\left(i\right)}$ and $DC_{\left(i\right)}$) by considering its WPT slotframe schedule. In addition, $PC_{\left(i\right)}$ and $DC_{\left(i\right)}$ are represented as follows:

(20) $$PC_{\left(i\right)}=\left[pc_{\left(i,1\right)},pc_{\left(i,2\right)},\cdots ,pc_{\left(i,k\right)}\right],\;\;\;(0<i\leq n_{node,j},\;0<j\leq M,\;0\leq k\leq l_{WPT,j}),$$

(21) $$DC_{\left(i\right)}=\left[dc_{\left(i,1\right)},dc_{\left(i,2\right)},\cdots ,dc_{\left(i,l\right)}\right],\;\;\;(0<i\leq n_{node,j},\;0<j\leq M,\;0\leq l\leq l_{WPT,j}),$$

where $pc_{\left(i,k\right)}$ and $dc_{\left(i,l\right)}$ represent cells (i.e., timeslot and channel offset) for energy harvesting and data transmission, respectively. Moreover, *k* and *l* refer to the cell index in $PC_{\left(i\right)}$ and $DC_{\left(i\right)}$. Note that $PC_{\left(i\right)}$ and $DC_{\left(i\right)}$ consist of at least $n_{reqPC,i}$ and $n_{reqDC,i}$. When the configuration of $PC_{\left(i\right)}$ and $DC_{\left(i\right)}$ is completed, the HAP transmits the modified 6P ADD response to the *i*-th cluster member.

**Figure 5:**
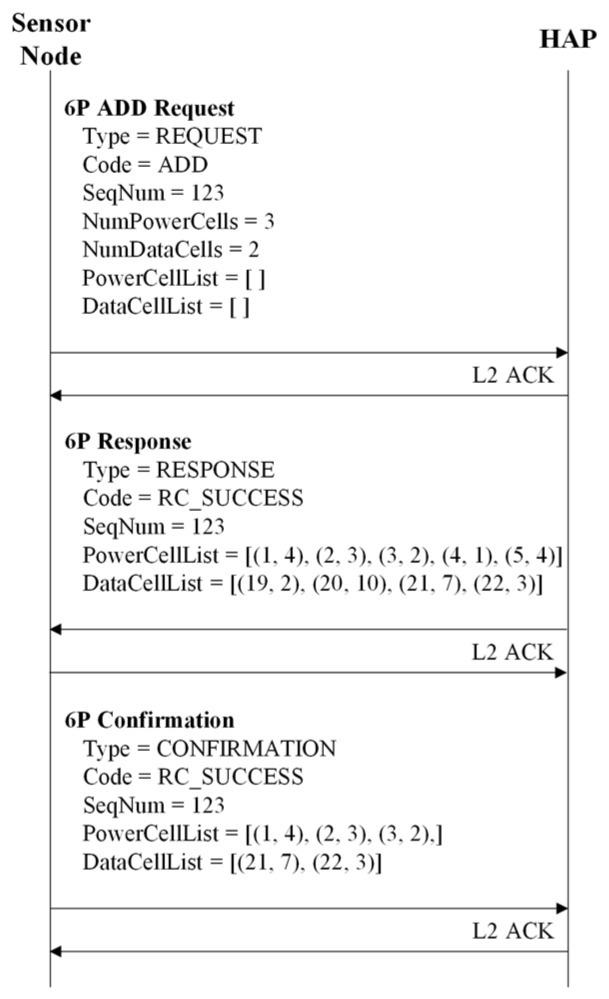
Example of the three-step 6P transaction.

Upon receiving the modified 6P ADD response, the *i*-th cluster member selects the required power and data cells from $PC_{\left(i\right)}$ and $DC_{\left(i\right)}$. Then, the *i*-th cluster member transmits the modified 6P ADD confirmation to its parent, including the selected required power and data cells. Finally, HAP adds the selected required power and data cells to its WPT slotframe schedule. Likewise, the *i*-th cluster member adds them to its WPT slotframe schedule. Thus, the three-step 6P transaction is completed. Figure 5 presents an example of the three-step 6P transaction in MCSS.

## 4. Performance Evaluation

The experimental simulation was conducted under the IEEE 802.15.4 physical (PHY)/MAC layer environment using the MATLAB simulator to evaluate the performance of MCSS. The simulation results of MCSS were compared to those of the legacy TSCH MAC [26] with a single slotframe and TMSS [31] to verify the superiority of MCSS. We describe the simulation configuration in the following subsections and discuss the simulation results in detail.

### 4.1. Simulation Configuration

In the simulation, we considered a cluster-tree network topology consisting of five HAPs and multiple sensor nodes, in which one HAP is designated as the root, and the other HAPs maintain the same number of sensor nodes as the cluster members and operate as their CHs. The number of cluster members varies from one to 10 to investigate the performance of MCSS as the number of sensor nodes changes. Moreover, we assumed that each HAP is randomly placed within the communication range of one of the other adjacent HAPs, and each sensor node is randomly placed within a distance of 2 m from its CH.

To investigate the effect of setting the initial structure of the TSCH concurrent slotframe suitable for the network size and topology, we considered a static cluster-tree network environment without changes in the number of sensor nodes and traffic within the cluster. We further assumed that all sensor nodes maintain the same residual energy of 140 mA at the start of the simulation. The timeslot length was set to 10 ms, and we assumed that the legacy TSCH MAC uses a single slotframe with a fixed length of 100 timeslots.

For MCSS, the lengths of the CM slotframe and HAP slotframe were set to 331 and five timeslots, respectively. The CM slotframe length was set by referring to the default setting in the Contiki implementation [36], which is a representative TSCH+RPL network implementation, including the exchange of CMs. The HAP slotframe length was empirically set to a prime number that maximizes the aggregate throughput through experiments.

The WPT slotframe length was determined by Algorithm 1, and its maximum value is limited to 101 timeslots, the nearest prime number to the length of a single slotframe of the legacy TSCH MAC (i.e., 100). If no limitation exists on the WPT slotframe length, then the number of sensor nodes that can be accommodated in the network is almost the same; thus, no meaningful change in the network performance can be obtained.

TMSS uses an EB slotframe for transmitting EB frames and CMs and a TMSS slotframe for energy harvesting and the data transmission of sensor nodes. Thus, in TMSS, the EB slotframe length was set to 331 timeslots, the same as the CM slotframe of MCSS, and the TMSS slotframe length was set to 100 timeslots considering the transmission period of data packets.

Moreover, each sensor node was assumed to transmit a certain number of data packets per second to its CH, set to one, two, and four. The data packet size and data rate were set to 127 bytes and 250 Kbps, respectively. The simulation parameters, such as the Tx power ($P_{Tx}$) for each HAP, path-loss parameter (α), and energy harvesting efficiency factor (η), were set to 100 mW, 2.7, and 0.65, respectively, to calculate the number of power cells. In addition, the energy consumed when a sensor node is in *Tx* ($P_{tx}$), *Rx* ($P_{rx}$), idle ($P_{idle}$), and sleep ($P_{sleep}$) states during one timeslot was set to 20.98, 17.96, 0.001, and 0.001 mA, respectively.

The performance of MCSS in terms of average end-to-end delay, aggregate throughput, and average harvested energy was compared to those of the legacy TSCH MAC and TMSS. In addition, the effect of WPT on the aggregate throughput was investigated. The simulation was iterated 100 times. The detailed parameters are listed in Table 1.

### 4.2. Simulation Results

In MCSS, the WPT slotframe length is determined according to the numbers of required power and data cells, among which the number of overprovisioned cells depends on the length of the CM slotframe and HAP slotframe. In the simulation, we first set the CM slotframe length shorter than the EB transmission period to ensure that the EB frame is transmitted at least once within the EB transmission period. Then, we experimentally investigated the HAP slotframe length that maximizes the aggregate throughput. In this experiment, the HAP slotframe length varied from three to 20.

**Figure 6:**
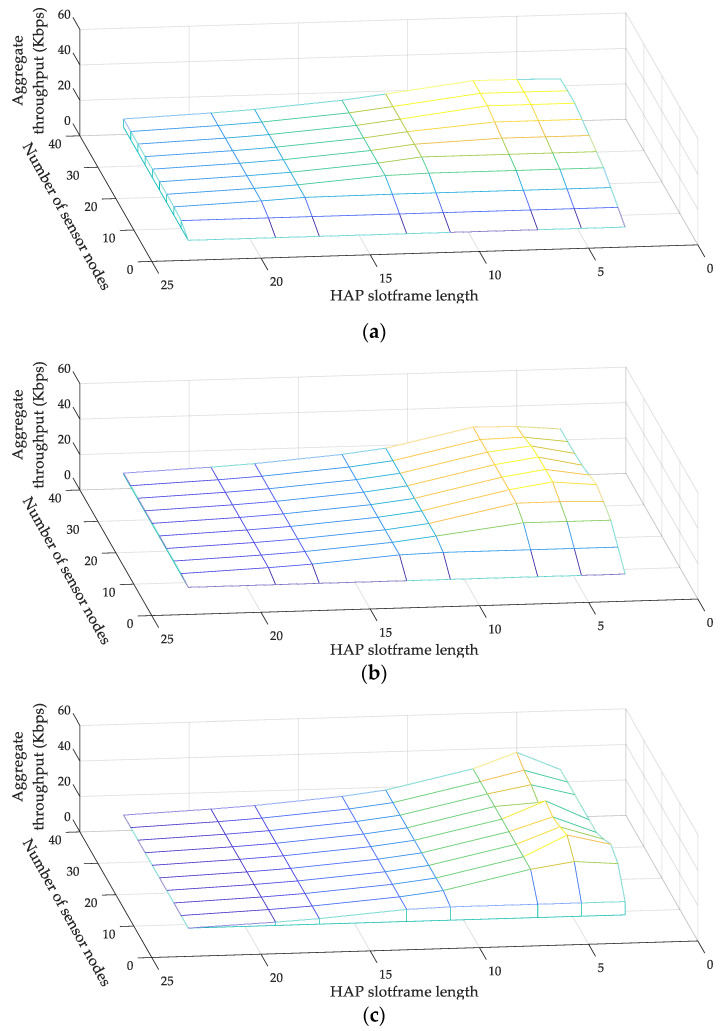
Aggregate throughput of MCSS: (**a**) one, (**b**) two, and (**c**) four packets/s.

Figure 6a–c illustrates the variations in the aggregate throughput of MCSS according to the change of the HAP slotframe length and the number of sensor nodes when the numbers of data packets transmitted per second are one, two, and four, respectively. Commonly in each figure, the aggregate throughput of MCSS increases as the HAP slotframe length decreases from 23 to five and decreases again when it changes from five to three. The shorter the HAP slotframe length, the more frequently data packets can be exchanged between HAPs.

However, the priority of a cell in the HAP slotframe is always higher than that of a cell in the WPT slotframe. Accordingly, when the HAP slotframe length is very short (i.e., three), the number of data packets transmitted from the sensor node to the CH in the WPT slotframe decreases, and as a result, the aggregate throughput decreases. In the figure, the color represents the level of the aggregate throughput; the brighter the color, the higher the aggregate throughput.

**Figure 7:**
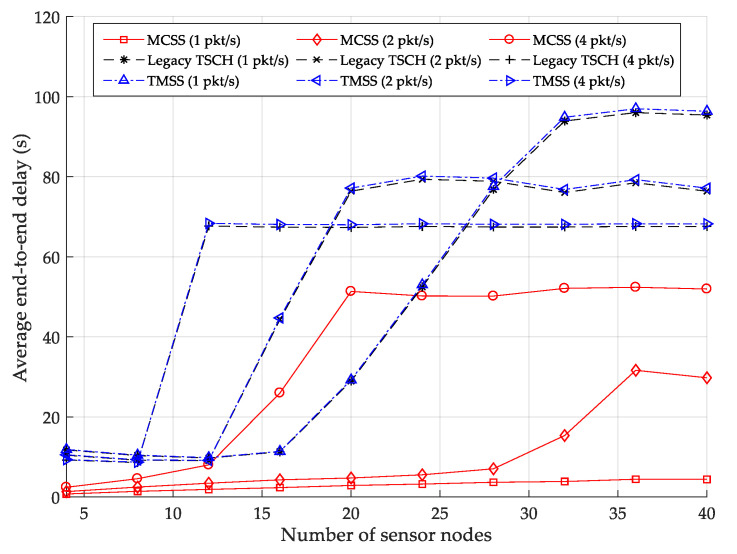
Average end-to-end delay.

As a result, when the HAP slotframe length is five, the overall aggregate throughput is the highest. Therefore, we set the HAP slotframe length to five in later experiments. Figure 7 illustrates the variations in the average end-to-end delay for various numbers of data packets transmitted per second. The average end-to-end delay indicates the average time taken by a data packet to be transmitted from the sensor node to the root. MCSS exhibits a lower average end-to-end delay than the legacy TSCH MAC and TMSS, regardless of the number of data packets transmitted per second. In MCSS, each HAP transmits data packets received from its cluster members to the root using the HAP slotframe, which is shorter than the TSCH slotframe and TMSS slotframe.

In MCSS, when the number of data packets transmitted per second is one, the average end-to-end delay does not increase significantly as the number of sensor nodes increases. This outcome is because, in MCSS, the HAP slotframe can sufficiently accommodate data traffic using the HAP slotframe, and only data transmission delays due to the overlapped cells occur. When the numbers of data packets transmitted per second are two and four, the average end-to-end delay increases until the numbers of sensor nodes are 36 and 20, respectively. This result is because, even if the number of data packets transmitted from the sensor nodes to HAPs (i.e., intra-cluster traffic) increases, the number of cells exchanging data packets between HAPs does not increase.

In such a case, if the numbers of sensor nodes are greater than 36 and 20, all cells in the WPT slotframe are occupied. Additional data cells cannot be allocated to the WPT slotframe, and thus the end-to-end delay is no longer increased. In the legacy TSCH MAC, data cells are randomly allocated to a TSCH slotframe of fixed length. Accordingly, the HAP may wait for the next TSCH slotframe to transmit because, in the TSCH slotframe, the data cells used for inter-cluster traffic transmission may be allocated to an earlier timeslot compared with the data cells used for intra-cluster traffic transmission. The average end-to-end delay of TMSS is similar to that of the legacy TSCH MAC but slightly longer.

The lengths of the TMSS slotframe and TSCH slotframe are equal to 100 timeslots; thus, in both cases, the same number of power and data cells for inter-cluster traffic and intra-cluster traffic transmissions are allocated in one slotframe. However, in TMSS, due to the use of a separate EB slotframe, overlap between cells may cause the delay of data packets. Quantitatively, when the numbers of data packets transmitted per second are one, two, and four, the average end-to-end delays of MCSS are 94.09% and 94.15%, 80.40% and 80.60%, and 37.41% and 38.03% shorter than those of the legacy TSCH MAC and TMSS, respectively.

**Figure 8:**
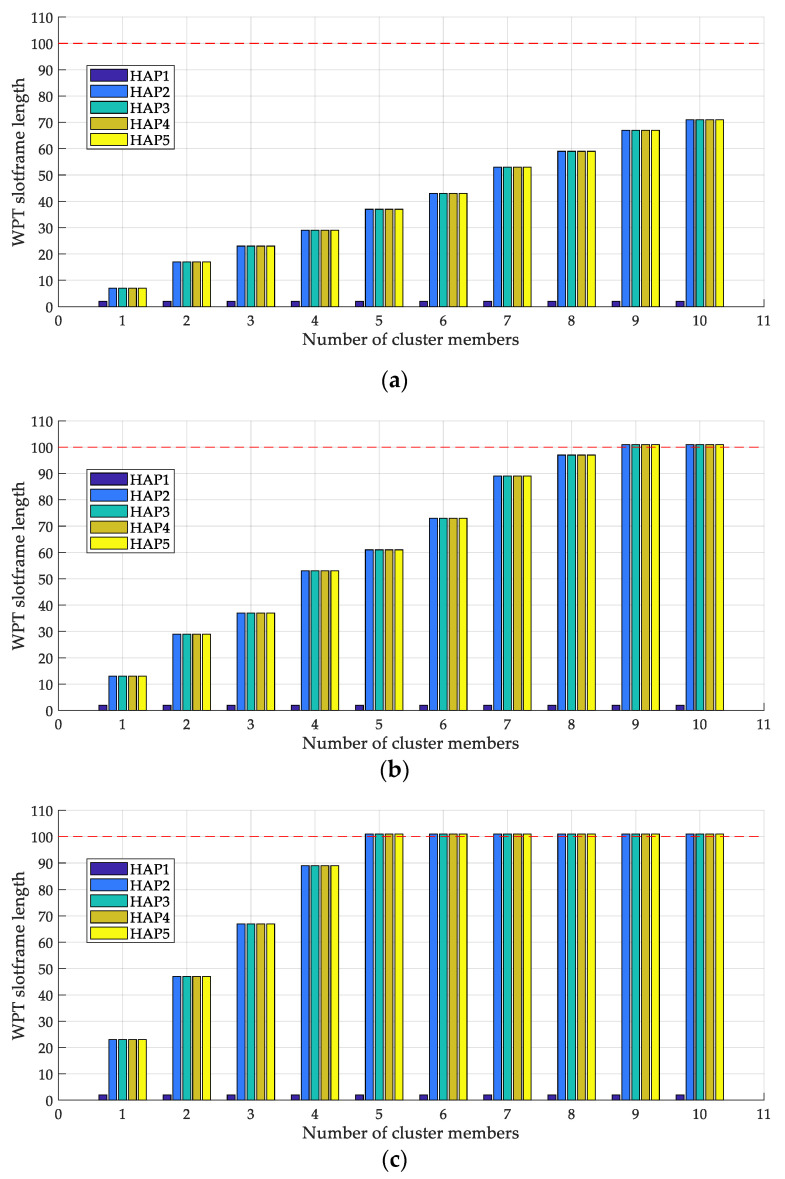
WPT slotframe lengths of HAPs: (**a**) one, (**b**) two, and (**c**) four packets/s.

Figure 8a–c illustrates the variations in the WPT slotframe length for each HAP according to the change in the number of cluster members for one, two, and four data packets transmitted per second, respectively. Commonly in each figure, the WPT slotframe length of HAP 1 equals two because HAP 1 is the root and does not have any cluster members. When the number of data packets transmitted per second is one, the WPT slotframe length of HAPs (except HAP 1) increases as the number of cluster members increases because the WPT slotframe length is determined to accommodate the increased power and data traffic in the HAP cluster. In contrast, when the number of data packets transmitted per second is two or four, it increases and is limited to 101, the maximum value.

**Figure 9:**
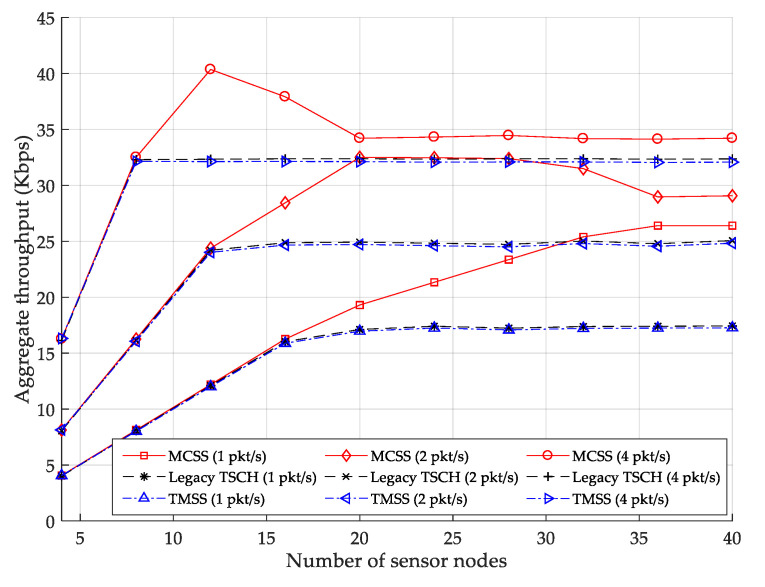
Aggregate throughput.

Figure 9 displays the change in the aggregate throughput as the number of data packets transmitted per second varies. In MCSS, only the data and power cells are allocated to the WPT slotframe; however, in the existing TSCH, all types of cells are allocated to one TSCH slotframe. Similarly, in TMSS, all power and data cells except for EB frames and CMs are allocated to the same TMSS slotframe. In other words, the WPT slotframe of the MCSS can accommodate more data cells of sensor nodes than the slotframe of the legacy TSCH MAC.

Therefore, in the figure, MCSS obtains a higher aggregate throughput than the legacy TSCH MAC and TMSS when the number of sensor nodes is larger than a certain number and the number of data packets transmitted per second is fixed. If the number of sensor nodes is less than a certain number, the aggregate throughput of all protocols is the same because their slotframe accommodates all network traffic. Quantitatively, when one, two, and four data packets are transmitted per second, the aggregate throughputs of MCSS are 26.82% and 26.60%, 18.63% and 18.37%, and 8.21% and 7.87% higher than those of the legacy TSCH MAC and TMSS, respectively.

**Figure 10:**
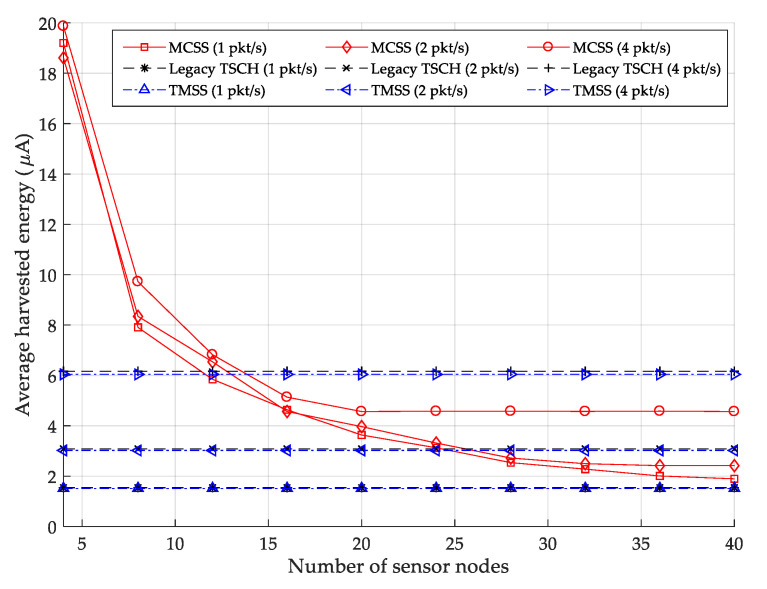
Average harvested energy.

Figure 10 depicts the average harvested energy for varying numbers of data packets transmitted per second. When the number of data packets transmitted per second is one, MCSS exhibits higher average harvested energy than legacy TSCH MAC and TMSS regardless of the number of sensor nodes because the WPT slotframe length is always shorter than the slotframe lengths of legacy TSCH MAC and TMSS.

When the number of packets transmitted per second is two or four, and if the number of sensor nodes is greater than 24 or 12, then the average harvested energy of the MCSS is lower than those of the legacy TSCH MAC and TMSS, as illustrated in Figure 8. Additionally, MCSS can accommodate power and data traffic for a larger number of sensor nodes than the legacy TSCH MAC and TMSS by using multiple concurrent slotframes. However, such multiple concurrent slotframes causes overlap between cells, thus, reducing the average energy harvested by individual sensor nodes in MCSS compared to the legacy TSCH MAC and TMSS.

When the number of data packets transmitted per second is one, the average harvested energy of MCSS decreases as the number of sensor nodes increases because an increase in the number of sensor nodes causes a decrease in the number of power cells for each sensor node allocated to the WPT slotframe. If the number of data packets transmitted per second is two or four, the average harvested energy of MCSS decreases only until it reaches a certain level. The reason is that the number of power cells successfully allocated to the WPT slotframe does not increase when the number of sensor nodes exceeds a certain number.

In contrast, the legacy TSCH MAC and TMSS maintain a constant average harvested energy that is almost similar to each other regardless of the number of sensor nodes because the number of power cells for each sensor node allocated to the slotframe is the same. In TMSS, the use of a separate EB slotframe enables energy harvesting of more sensor nodes compared to the legacy TSCH MAC. However, TMSS exhibits a slightly lower average harvested energy than the legacy TSCH MAC due to the overlap between cells. When the numbers of data packets transmitted per second are one, two, and four, the average harvested energies of MCSS are 244.30% and 244.28%, 79.68% and 79.68%, and 11.90% and 11.90% higher than those of the legacy TSCH MAC and TMSS, respectively.

**Figure 11:**
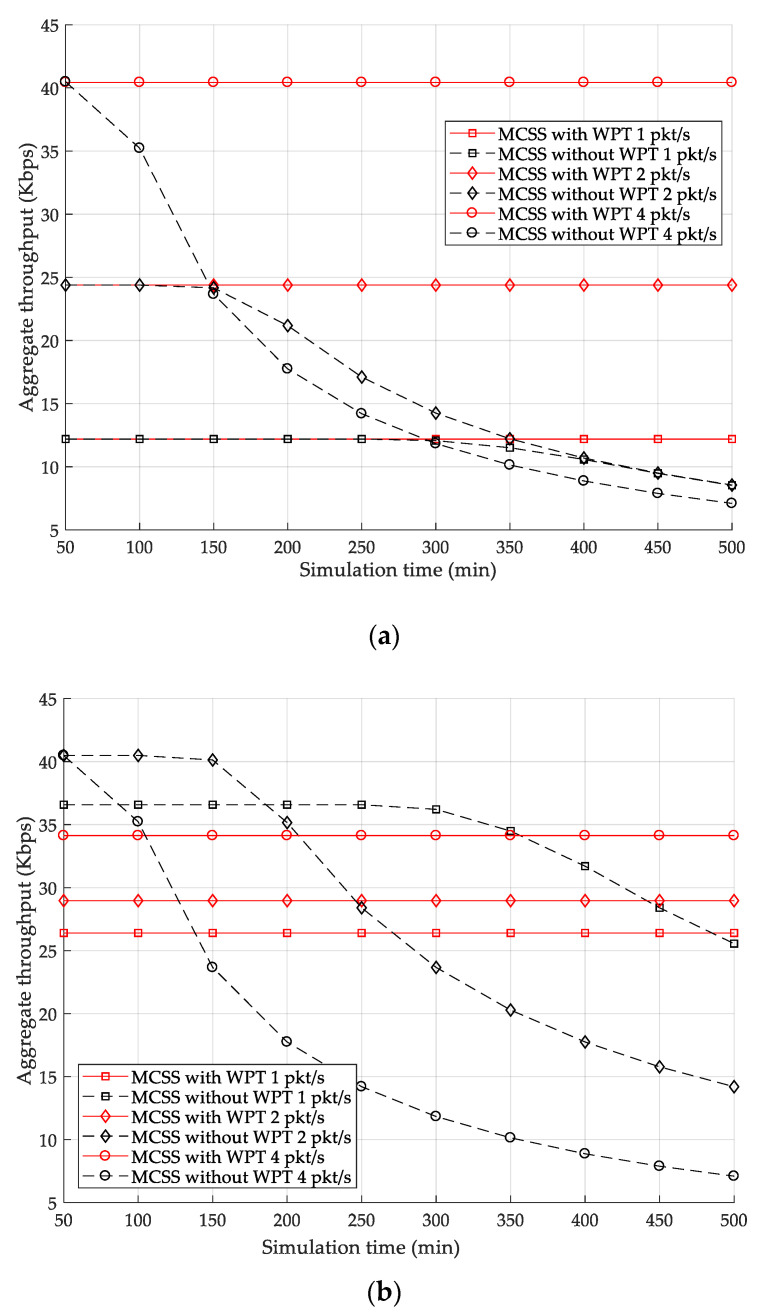
Effect of WPT on aggregate throughput: (**a**) 12 and (**b**) 36 sensor nodes.

To investigate the effect of WPT on the performance of MCSS, we compared the aggregate throughput for the case where the HAP transmits power to the sensor nodes (i.e., MCSS with WPT) and the case where it does not (i.e., MCSS without WPT). In this experiment, the initial residual energy of sensor nodes was randomly set between 70 and 140 mA, and the simulation time increased from 50 to 500 min in 50 min increments. Figure 11a,b illustrates the variations in the aggregate throughput of MCSS for varying simulation times when the numbers of sensor nodes are 12 and 36, respectively.

In the case of MCSS with WPT, the aggregate throughput is maintained as constant regardless of the simulation time because sensor nodes perform energy harvesting so that the energy-depleted dead nodes do not occur. On the other hand, as the simulation time increases, the aggregate throughput in the case of MCSS without WPT remains constant until a certain simulation time, and then starts to decrease. In the part in which the aggregate throughput is maintained constant, the dead nodes do not occur because the amount of energy consumed by the sensor nodes is less than their remaining energy. In contrast, in the opposite case, as the simulation time increases, the number of dead nodes increases, and, as a result, the aggregate throughput decreases.

In Figure 11a, in both cases, all sensor nodes successfully allocate the required data cells in the WPT slotframe; thus, the aggregate throughput remains the same until a certain simulation time. On the other hand, in Figure 11b, in the case of MCSS with WPT, there are sensor nodes that cannot allocate their own data cells due to the increase of the number of power cells allocated in the WPT slotframe; thus, there is a part in which the aggregate throughput in the case of MCSS without WPT is higher than that in the case of MCSS with WPT. However, in the case of MCSS without WPT, the number of dead nodes increases as the simulation time increases. Accordingly, after a certain simulation time, the aggregate throughput of MCSS without WPT becomes lower than that of MCSS with WPT.

## 5. Conclusions

This paper presented an MCSS protocol designed to support a cluster-tree network topology and various traffic types in WPT-enabled WSNs. In addition, MCSS defines three types of TSCH concurrent slotframes: CM slotframes, HAP slotframes, and WPT slotframes, to schedule the CM traffic, inter-cluster traffic, and intra-cluster traffic for WPT-enabled WSNs, respectively. To address the problems caused by overlapped cells, MCSS sets each slotframe to a different length and transmission priority.

In particular, the WPT slotframe length for each cluster was determined based on the total number of required power and data cells, including the calculated number of overprovisioned cells. The experimental simulation was conducted in an environment of varying network sizes. The performances of MCSS, legacy TSCH MAC, and TMSS were compared in terms of the average end-to-end delay, aggregate throughput, and average harvested energy to demonstrate the superiority of MCSS.

## Figures and Tables

**Table 1:** Simulation parameters.

Parameter	Value	Parameter	Value
PHY/MAC	IEEE 802.15.4	*d*	0–2 m
Number of sensor nodes	4–40	Data packets transmitted per second	1, 2, 4
Packet size	127 bytes	$P_{tx}$	20.98 mA
Data rate	250 Kbps	$P_{rx}$	17.96 mA
Timeslot length	10 ms	$P_{idle}$	0.001 mA
CM slotframe length	331	$P_{sleep}$	0.001 mA
HAP slotframe length	5	$P_{Tx}$	100 mW
WPT slotframe length	2–101	α	2.7
TSCH slotframe length	100	η	0.65

## Data Availability

No new data were created or analyzed in this study. Data sharing is not applicable to this article.

## Abbreviations

The following abbreviations are used in this manuscript:

6P	6top Protocol
6TiSCH	IPv6 over the TSCH mode of the IEEE 802.15.4e
6top	6TiSCH Operation Sublayer
Ack	Acknowledgment
CH	Cluster Head
CM	Control Message
CSMA/CA	Carrier Sense Multiple Access with Collision Avoidance
DIO	DODAG Information Object
DODAG	Destination-Oriented Directed Acyclic Graph
EB	Enhanced Beacon
HAP	Hybrid Access Point
IoT	Internet of Things
LCM	Least Common Multiple
MAC	Medium Access Control
MCSS	Multiple Concurrent Slotframe Scheduling
OF	Objective Function
PHY	Physical
PRU	Power Receiving Unit
PTU	Power Transmitting Unit
RF	Radio Frequency
RPL	Routing Protocol for Low-power and Lossy Networks
RSSI	Received Signal Strength Indicator
Rx	Reception
TDMA	Time-Division Multiple Access
TMSS	TSCH Multiple Slotframe Scheduling
TSCH	Time-Slotted Channel Hopping
Tx	Transmission
WPT	Wireless Power Transfer
WSN	Wireless Sensor Network
